# Role of COVID-19 Vaccine in the Management of Gynecologic Oncology Lymphadenopathies

**DOI:** 10.3390/ijerph21081063

**Published:** 2024-08-14

**Authors:** Laura Fernandez Sanahuja, Ester Miralpeix, Josep Maria Solé Sedeño, Marta Baucells, Berta Fabregó, Ana Sierra, Gemma Mancebo

**Affiliations:** 1 Department of Obstetrics and Gynecology, Hospital del Mar, 08003 Barcelona, Spain; lfernandezsanahuja@psmar.cat (L.F.S.); emiralpeix@psmar.cat (E.M.); jsole@psmar.cat (J.M.S.S.); mbaucells@psmar.cat (M.B.); bfabrego@psmar.cat (B.F.); 2 School of Medicine, Universitat Pompeu Fabra, 08003 Barcelona, Spain; 3 Department of Radiology, Hospital del Mar, 08003 Barcelona, Spain; amsierra@psmar.cat

**Keywords:** COVID-19, vaccination, gynecological cancer, lymphadenopathies

## Abstract

Background: This study aimed to evaluate the incidence of lymphadenopathies after COVID-19 vaccination and their impact on the clinical management of gynecologic oncology patients. Methods: A retrospective observational study was conducted involving patients who underwent abdominopelvic or thoracoabdominopelvic CT scans during diagnosis or follow-up. Patients were classified into a vaccinated group (Vac group) and a non-vaccinated group (NoVac group). The radiological appearance of lymphadenopathies was categorized as low or high risk of malignancy, and management strategies were recorded as standard management or additional assessment. Results: 75 patients were included, with 44 in the Vac group and 31 in the NoVac group. The incidence of lymphadenopathies was similar between the groups: 34.1% in the Vac group and 32.3% in the NoVac group (*p* = 0.868). High-risk lymphadenopathies were observed in 20.4% of the Vac group and 22.6% of the NoVac group, while low-risk lymphadenopathies were seen in 13.6% of the Vac group and 9.7% of the NoVac group (*p* = 0.691). Standard management was the most common approach, used in 80.0% of the Vac group and 100.0% of the NoVac group (*p* = 0.25). Conclusions: COVID-19 vaccine does not increase the incidence of lymphadenopathies in imaging tests of gynecological cancer patients.

## 1. Introduction

The SARS-CoV-2 (COVID-19) pandemic has represented an immense challenge for global health systems. Adapting to this new situation has significantly impacted the management of gynecologic oncology patients, prompting the development of optimized surveillance protocols and the integration of telemedicine and shared decision-making [1,2]. The pandemic has underscored the necessity of flexibility and innovation in patient care, leading to advancements in remote consultations and the reevaluation of routine practices.

Although the efficacy of COVID-19 vaccines is well established, with substantial evidence supporting their role in preventing severe disease and transmission, some studies have associated vaccination status with lymphadenopathies [3,4,5,6,7,8]. This association has emerged as a notable concern, particularly in the context of cancer patients who undergo regular imaging studies for disease monitoring. The incidence of lymphadenopathies following COVID-19 vaccination has been reported to be around 11.6% and 16% [9]. These lymphadenopathies are predominantly observed in lymph nodes ipsilateral to the vaccine injection site. Specifically, axillary, interpectoral, supraclavicular, and lower cervical lymphadenopathies have been identified as common side effects, particularly after the second dose of the vaccine [4,10,11]. This observation is significant as it potentially overlaps with lymph node territories frequently evaluated in oncological follow-up.

Moreover, there have been reports of rare cases involving iliac and inguinal lymphadenopathies following vaccination when the injection site is in the ipsilateral thigh muscle. Additionally, systemic lymphadenopathies have been documented after deltoid muscle injections, although these are less common [12,13]. These findings highlight the need for a comprehensive understanding of the range and locations of lymphadenopathies associated with COVID-19 vaccination to prevent misinterpretation during cancer surveillance.

Regarding the size and duration of the affected lymphadenopathies, the lymph nodes usually measure between 1 and 4 cm. The swelling typically appears within a few days after vaccination and can persist for several weeks [9]. This transient nature of the lymphadenopathy complicates the differentiation between vaccine-related and pathology-related lymph node enlargement. As a result, there has been a significant focus on managing these effects in patients undergoing cancer treatment, especially in those with existing lymphatic concerns.

Recent research has predominantly centered on breast cancer, hematological malignancies, and lung cancer survivors, due to the anatomical location and high frequency of lymph node involvement in these cancers [5,8,10,11]. These studies have highlighted the importance of distinguishing between reactive lymphadenopathies caused by the vaccine and those indicative of disease progression. Some authors have suggested postponing routine imaging studies for up to 6 weeks after COVID-19 vaccination to allow for the resolution of reactive lymphadenopathies, thereby avoiding unnecessary diagnostic confusion [4,8].

In January 2021, Spain launched a national COVID-19 vaccination program, initially targeting elderly individuals, very high-risk patients, and healthcare workers, followed by the general population. By September 2021, approximately 77% of the population in Spain had received at least one dose of the COVID-19 vaccine [14]. Despite the high vaccination coverage, the precise impact of COVID-19 vaccination on lymphadenopathies in the context of gynecologic oncology diagnosis and surveillance remains unclear. The lymphatic drainage from the arm typically affects axillary and supraclavicular lymph nodes, which are not the primary focus in gynecologic oncology patients. Consequently, there is a lack of published studies evaluating the impact of COVID-19 vaccines on the management of gynecologic oncology patients.

Given these considerations, the present study was designed to fill this gap by evaluating the incidence of lymphadenopathies related to COVID-19 vaccination specifically in gynecologic oncology patients. The study aims to assess the implications of these findings for clinical practice and determine whether modifications in clinical practice or follow-up protocols are warranted in the context of COVID-19 vaccination. By addressing these questions, our research seeks to contribute valuable insights into the optimal management of gynecologic oncology patients amid the ongoing pandemic, ensuring that their care remains effective and aligned with the evolving understanding of COVID-19-related side effects.

This study not only provides important data on the occurrence of lymphadenopathies post-vaccination, but also informs best practices for integrating vaccination history into patient management plans. It is crucial that future research continues to explore this area to further refine our approaches and ensure the best outcomes for patients navigating both cancer and vaccination considerations.

## 2. Materials and Methods

This retrospective observational study was conducted in a single tertiary care institution from January 2021 to August 2021 in the Multidisciplinary Unit of Gynecological Tumors in Hospital del Mar, Barcelona, Spain.

All patients who underwent an abdominopelvic or thoracoabdominopelvic computed tomography (CT) scan either at diagnosis or follow-up for a gynecological cancer were included. The exclusion criteria were patients under 18 years of age; lack of histological confirmation for gynecological cancer; concomitant malignancies; patients under immunosuppressive treatment, autoimmune diseases, or with any congenital or acquired immunodeficiency; and unavailability or absence of medical records.

Patients were classified into the vaccinated group (Vac group), which included those who received almost one vaccine dose before the CT scan, and the non-vaccinated group (NoVac group), which included non-vaccinated patients at the time of the CT scan.

Lymphadenopathies were stratified as low or high risk of malignancy according to radiological criteria. Lymphadenopathies less than 1 cm and no change compared to previous CT scans were classified as having a low risk of malignancy. Those greater than 1 cm in diameter, with infiltration of surrounding tissues or growth compared to CT scan controls, were considered to have a high risk of malignancy [15].

The locations of the lymphadenopathies were also recorded, with four possible sites: the supradiaphragmatic site, which included axillar, intrathoracic, and supraclavicular lymphadenopathies; the retroperitoneal site, which included paraaortic and pelvic lymphadenopathies; the inguinal site; and finally, other sites.

The incidence and type of lymphadenopathies were compared between the Vac and NoVac groups. Additionally, the management plans based on these findings were compared, which could include additional assessment when another test was performed to confirm the suspected diagnosis (advanced imaging control, additional imaging test or invasive test) or standard management when no additional assessment was performed because presumptive diagnosis was given by the clinical context.

Categorical variables are reported as frequencies and percentages. Continuous variables are reported as medians and standard deviations (SDs). Pearson’s chi-square test or Fisher’s exact test was used to compare categorical variables. These tests were chosen for their robustness in assessing associations between categorical variables. For continuous variables, Student’s *t*-test was utilized to compare means between the two groups. This test is robust for normally distributed data, and we verified normality using the Shapiro–Wilk test. For continuous variables that did not meet the normality assumption, we employed the Mann–Whitney U test, a non-parametric alternative that does not assume a normal distribution, ensuring the robustness of our comparisons. All the statistical tests were performed using IBM SPSS Statistics Version 28 (IBM Corporation, Armonk, NY, USA) and were two-sided, and *p* < 0.05 was considered to indicate statistical significance.

## 3. Results

**Figure 1:**
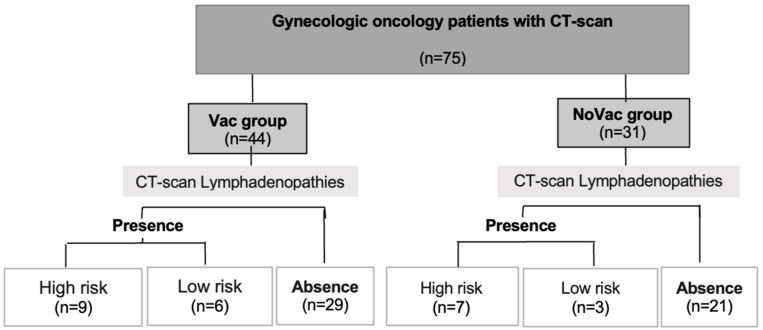
Study cohort.

In the final analysis, 75 patients were included. Of these, 44 patients were classified in the Vac group and 31 were classified in the NoVac group (Figure 1).

The demographic data of the patients are presented in Table 1. The mean age was 70.6 years (SD 11.2 years) in the Vac group, and 61.2 years (SD 14.9 years) in the NoVac group (*p* = 0.003). The most frequent neoplasms were endometrial (41.3%) and ovarian (40.0%) tumors. Of the 44 patients vaccinated, 22 (50.0%) received Pfizer-BioNTech, 5 (11.4%) received AstraZeneca, 16 (36.6%) received Moderna, and 1 (2.3%) received Jansen. The median time between vaccination and CT scan was 3.5 weeks (SD: 4.5).

The characteristics of the patients with lymphadenopathies are described in Table 2. Lymphadenopathies were identified via CT scan in 25 of 75 patients (33.3%), 15 patients (34.1%) in the Vac group and 10 patients (32.3%) in the NoVac group (*p* = 0.868). In the Vac group, 9 of 44 patients (20.4%) presented high-risk lymphadenopathies in the imaging test, whereas 6 of 44 patients (13.6%) presented a low risk of malignancy lymphadenopathies. On the other hand, in the NoVac group, 7 of 31 patients (22.6%) presented a high risk of malignancy lymphadenopathies and 3 of 31 patients (9.7%) presented a low risk of malignancy lymphadenopathies. No statistically significant differences between groups were observed (*p* = 0.691).

Most of the 25 patients with lymphadenopathies on the CT scan (84.0%) did not require any additional tests to confirm malignancy, and there were no statistically significant differences between the two groups (80.0% in the Vac group versus 100.0% in the NoVac group, *p* = 0.25), as shown in Table 3. Of all 25 patients with lymphadenopathies, 3 (12%) required additional assessment due to high-risk lymphadenopathies on the CT scan, and all were in the Vac group. In one of these three patients, PET-CT was performed to confirm the relapse of her ovarian cancer. In another patient, an inguinal lymphadenopathy was observed on the CT scan for cervical cancer follow-up, and a biopsy confirmed malignancy. Finally, the last case in which an additional assessment was performed was a patient whose CT scan, after 13 months of follow-up for endometrial cancer, demonstrated cancer progression with pulmonary metastasis and growth of the paraaortic and pelvic lymphadenopathies. These 3 patients and 22 other patients with radiological lymphadenopathies are extensively described in Table S1.

Finally, the most common location of the lymphadenopathies was retroperitoneal, being registered in 21 of the 25 patients with lymphadenopathies. Only six patients had inguinal lymphadenopathies, and five had supradiaphragmatic lymphadenopathies.

## 4. Discussion

To our knowledge, this is the first study to evaluate the incidence of lymphadenopathies after COVID-19 vaccination in patients with gynecologic cancer. Based on our results, it seems that the COVID-19 vaccine does not increase the incidence of lymphadenopathies in gynecologic oncology patients, and moreover it does not modify standard clinical practice.

Previous published studies suggest that COVID-19 vaccination can cause possible dilemmas in differentiating vaccine-related reactive lymphadenopathy from that related to malignant diseases [3,4,5,6,7,8]. In our study, the incidence of lymphadenopathies in gynecologic oncology patients after receiving at least one dose of the COVID-19 vaccine was 34.1%, which was not statistically significantly different from that in the NoVac group (32.3%; *p* = 0.868). This incidence was greater than that in the general population (11.6–16%) [9]. However, compared with those of patients who were primarily diagnosed with breast, lung, head and neck, skin, or hematological neoplasms, the incidence of lymphadenopathies after the COVID-19 vaccine ranged from 36.4% to 54% [3,11]. A study published by Cohen et al., which included patients with lymphoma, lung, or breast malignancy, revealed a statistically significant difference between the vaccinated groups and the control group (36.4% after one dose, 53.9% after two doses, versus 7.6% in the control group; *p* < 0.01) [3]. Another study by Skawran et al. compared the FDG-avidity of the axillary lymph nodes ipsilateral to COVID-19 vaccination via a PET-CT scan in 140 oncological patients who were mainly diagnosed with head and neck, lung, skin, or hematological neoplasms. In this study, increased avidity was identified in 54% of the subjects [11]. An important consideration when interpreting the data is the location of the lymphadenopathies. In our study, the most frequent location was retroperitoneal, which differs from other published studies that included patients with hematological neoplasms, breast, head and neck, or lung malignancies, where the most frequent locations of node metastasis were axillary, cervical, and intrathoracic [3,6,11]. Differences in the sites of lymphadenopathy can be explained by several molecular and biological factors. Gynecological organs have unique lymphatic drainage patterns. For example, tumors of the uterus and cervix tend to spread first to the pelvic lymph nodes due to their anatomical proximity and specific lymphatic drainage pathways. Additionally, tumor cells express adhesion molecules and chemokine receptors that facilitate their migration to specific sites. For instance, the expression of CCR7 in cervical cancer tumor cells can direct them toward lymph nodes that express CCL21, one of its chemokine ligands. This should be considered when interpreting the possible side effects of COVID-19 vaccines, in which the most frequent location of reactive lymphadenopathies is ipsilateral to vaccine inoculation, in the axilla and supraclavicular, interpectoral, and cervical regions [16]. In our study, no differences in the incidence of lymphadenopathies were observed between the Vac and NoVac groups, which may be explained by the fact that nodal territories affected by gynecological cancers differ from those affected by the COVID-19 vaccine.

Our analysis of the radiological appearance of lymphadenopathies revealed an incidence of 16.6% of low-risk malignancy lymphadenopathies in the Vac group. The incidence was much lower than that reported in previous studies of patients who were mainly diagnosed with breast, lung, head and lung, skin, or hematological neoplasms, which described 36.5% and 37%, respectively, of vaccine-associated lymphadenopathies in patients vaccinated against COVID-19 [3,11]. The lower incidence of low-risk lymphadenopathies reported in our study is probably because the inflammatory response to COVID-19 vaccination does not affect these node territories. On the other hand, our incidence of high-risk malignancy lymphadenopathies in vaccinated patients (20.4%) was greater than that reported by the previous authors, which varies between 2.3% and 6.7% [3,11]. The incidence observed in our study closely resembles the rates of positive lymphadenopathies found at the time of diagnosis and follow-up in endometrial and ovarian cancers. At the diagnosis of endometrial and ovarian cancer, the ranges of positive lymphadenopathy rates are 10–20% and 20–30%, respectively. During follow-up of endometrial and ovarian cancer, recurrent positive lymphadenopathies can occur in about 10–15% and 25–30% of patients, respectively, who initially had negative lymph nodes at diagnosis. This could explain the high rate reported [17,18]. In addition, the study published by Cohen et al. reported 2.3% of high-risk malignancy lymphadenopathies in vaccinated patients and 7.6% in the control group. These differences were statistically significant (*p* < 0.01), in contrast to our study, in which no statistically significant differences were detected [3]. These differences observed between the studies may be due to the location of the lymphadenopathies. In gynecological cancers, the nodal territories affected by cancerous cells differ from those that may be reactive to vaccination, so no statistically significant differences were found between the groups.

The assessment of clinical management after identifying lymphadenopathies is still controversial and depends on multiple factors, including previous vaccinations. In our study, the clinical management associated with lymphadenopathies and vaccination status did not differ between groups and decisions were made according to radiological and oncological criteria based on previous vaccination experience. Before COVID-19 vaccination, the appearance of lymphadenopathies had already been observed after receiving other vaccines, for example, against Human Papilloma Virus and Influenza Virus [19,20,21]. The proportion of lymphadenopathies secondary to these vaccines (4/83 for the Seasonal Influenza Virus vaccine and only a few cases described for the Human Papilloma Virus vaccine) is much lower than that observed with the COVID-19 vaccine (11.6–16%), and consequently no consideration has been given to modifying the diagnostic or follow-up protocols for oncology patients [9,19,20,21]. Apparently, other authors such as Skawran et al. reported that clinical assessment differed in 12% of patients with lymphadenopathies, mainly due to the use of additional sonography and biopsy to exclude malignancy. This may be explained by the fact that the oncologic history of the patients included melanoma, breast cancer, pharyngeal cancer, chronic lymphocytic leukemia, or lymphoma, all of which are neoplasms with a high risk of lymphadenopathy relapse. Moreover, in this study, the imaging technique used was PET-CT in consideration of [^18^F]FDG-uptake intensity to analyze and classify lymphadenopathies [11]. On the other hand, the study published by Lim et al. reported six cases of a lymphadenopathy finding after COVID-19 vaccination in breast cancer patients. Biopsy was performed in three patients, all of whom had benign hyperplasia, while the other three patients underwent 4–12 weeks of follow-up with imaging tests [10]. Faced with this controversy, some authors propose an additional evaluation at 4–12 weeks when lymphadenopathies after COVID-19 vaccination are found in patients with melanoma, breast, head and neck, or hematological cancer [22,23,24,25,26]. Other authors have proposed delaying radiologic studies 3–6 weeks after the administration of the vaccine in patients with breast, hematological, lung, and head and neck neoplasms to avoid increasing the number of unnecessary tests [3,22,23,24,27]. Our data do not support these recommendations for gynecologic cancer patients. Like other authors, we recommend collecting the vaccination status, date, and location of the vaccine injection when imaging studies are performed in oncological patients [3,4,10,23]. Even though it seems that it is not necessary to delay imaging tests in gynecologic cancer patients, or to perform additional evaluation, it is important for the oncology community to consider the potential implications of post-vaccination lymphadenopathies in patients undergoing cancer treatment or follow-up. The identification of lymphadenopathies can be a source of diagnostic confusion, potentially leading to unnecessary biopsies or alterations in therapeutic strategies. Awareness of this phenomenon can assist clinicians in differentiating between vaccine-induced lymphadenopathy and disease progression or recurrence, thereby avoiding unwarranted diagnostic procedures and anxiety for patients. It is essential to maintain clear communication with patients to explain the benign nature of these post-vaccination lymphadenopathies and thus avoid unnecessary concerns. In the absence of worrying symptoms or evidence of disease progression, no additional specific interventions are required beyond usual clinical practices.

This study has several strengths that deserve to be mentioned. To our knowledge, this is the first study assessing the role and impact of the COVID-19 vaccine in gynecological oncology patients and the management of their lymphadenopathies. Additional strengths include the homogeneous nature of the study population, in terms of both demographics and clinical characteristics. Another strength of the study is the homogeneity of the multidisciplinary team, with consistent professionals following the same criteria and protocols throughout. Conversely, potential weaknesses of our study include the non-randomized control trial design and the inherent limitations and bias associated with a retrospective study. This could be addressed in future research by implementing a prospective study design that would allow more controlled and systematic data collection, reducing the potential for bias inherent in retrospective studies. However, the enrolment of all consecutive patients reduced the chance of selection bias, and clinical stage and comorbidities were well matched between groups. In addition, the limited number of patients and the single-center design may have limited the potential extrapolation of our results or resulted in statistically significant differences. This could be addressed by collaborating with multiple centers, providing a larger and more diverse patient population that would enhance the external validity of the study. Finally, a joint analysis of the different gynecological neoplasms was conducted given the limited sample size available. However, we believe this does not impact the main objective of our study, which is the detection of radiological lymphadenopathies, as most gynecological neoplasms can affect similar lymph node territories.

## 5. Conclusions

In conclusion, our study suggests that COVID-19 vaccination does not appear to increase the incidence of lymphadenopathies observed in imaging tests of gynecological cancer patients. Additionally, there is no evidence to indicate that the vaccination has a significant impact on clinical oncological management. These findings are crucial as they provide reassurance to both patients and healthcare providers that the COVID-19 vaccination does not interfere with the diagnostic processes or treatment plans for gynecological cancers. Consequently, based on our results, it is not necessary to adjust the scheduling of imaging tests based on the timing of COVID-19 vaccinations.

However, it is still advisable to document vaccination status in patient medical records, including the date and location of the vaccine injection. Accurate record-keeping can help differentiate between vaccine-related and disease-related lymphadenopathy, should any concerns arise during clinical evaluations. This practice ensures that all healthcare professionals involved in the patient’s care are aware of their vaccination history, which can be important for interpreting imaging results and managing patient care effectively.

Although our study indicates that COVID-19 vaccination does not seem to significantly impact the incidence of lymphadenopathies, there are several areas where further research is warranted. First, additional studies focused specifically on gynecological cancer populations are needed to confirm and expand upon these findings. Such studies should aim to include larger sample sizes and diverse patient cohorts to enhance the generalizability and robustness of the results.

Moreover, it could be valuable to investigate how post-vaccination lymphadenopathy affects the quality of life of patients with gynecological cancers. This includes exploring potential psychological impacts, such as anxiety and stress, which may be associated with the occurrence of lymphadenopathy in this context. Understanding the psychological burden of vaccine-related lymphadenopathy can help in developing supportive interventions to address these concerns and improve patient well-being.

Future research should also explore the potential for any long-term effects or delayed reactions related to COVID-19 vaccination and how these may influence cancer management and patient outcomes. Studies could examine whether there are specific patterns or types of lymphadenopathy that might be more likely to occur in certain subgroups of patients or whether there are differences based on the type of COVID-19 vaccine administered.

Additionally, research into the interactions between COVID-19 vaccination and other therapeutic interventions for cancer, such as chemotherapy or targeted therapies, could provide valuable insights. Understanding these interactions will be crucial for optimizing patient management strategies and ensuring that the benefits of vaccination are maximized while minimizing any potential negative impacts on cancer treatment.

In summary, while our findings suggest that COVID-19 vaccination does not have a significant impact on the incidence of lymphadenopathies or clinical management of gynecological cancer, there remains a need for continued research in this area. By focusing on the psychological and long-term effects of post-vaccination lymphadenopathy, as well as its potential interactions with cancer therapies, future studies can contribute to a more comprehensive understanding of the implications of COVID-19 vaccination for patients with gynecological cancers. We recommend that future research prioritizes these areas to enhance the overall management and quality of care for this patient population in the context of ongoing vaccination efforts.

## Figures and Tables

**Table 1:** Demographic and clinical characteristics of the sample (n = 75).

	**Total** (n = 75)	**Vac Group** (n = 44)	**NoVac Group** (n = 31)	***p* Value**
**Patient age** (y, $\bar{x}$ ± s)	65.1 ± 13.5	70.6 ± 11.2	61.2 ± 14.9	0.003 ^a^
**Type of neoplasm** (n, %)				0.496 ^c^
- Ovarian	30 (40.0)	21 (47.7)	9 (29.0)	
- Endometrium	31 (41.3)	17 (38.6)	2 (6.5)	
- Uterine sarcoma	3 (4.0)	1 (2.3)	14 (45.2)	
- Uterine cervix	9 (12.0)	4 (9.1)	5 (16.1)	
- Vulva	2 (2.7)	1 (2.3)	1 (3.2)	
**CT scan** (n, %)				0.544 ^b^
- Staging	19 (25.3)	9 (20.5)	10 (32.3)	
- Follow-up	56 (74.7)	35 (79.5)	21 (67.7)	
**COVID-19 infection** (n, %)				0.638 ^c^
- Yes	4 (5.3)	3 (6.8)	1 (3.2)	
- No	71 (94.7)	41 (93.2)	30 (96.8)	

^a^ = Student’s *t*-test; ^b^ = Pearson’s chi-square test; ^c^ = Fisher exact test; *p* < 0.05 was considered significant.

**Table 2:** Lymphadenopathies characteristics detected by CT scan (n = 75).

	**Total** (n = 75)	**Vac Group** (n = 44)	**NoVac Group** (n = 31)	***p* Value**
**Lymphadenopathies** (n, %)				
- Presence	25 (33.3)	15 (34.1)	10 (32.3)	0.868 ^a^
- Absence	50 (66.7)	29 (65.9)	21 (67.7)	
**Lymphadenopathies characteristics on CT scan** (n, %)				
- High-risk	16 (21.3)	9 (20.4)	7 (22.6)	0.691 ^b^
- Low-risk	9 (12.0)	6 (13.6)	3 (9.7)	

^a^ = Pearson’s chi-square test; ^b^ = Fisher exact test; *p* < 0.05 was considered significant.

**Table 3:** Clinical management according to COVID-vaccine status of patients with lymphadenopathies on the CT scan (n = 25).

	**Total** (n = 25)	**Vac Group** (n = 15)	**NoVac Group** (n = 10)	***p* Value**
**Clinical management** (n, %)				
- Standard management	22 (88.0)	12 (80.0)	10 (100.0)	0.25 ^a^
- Additional assessment	3 (12.0)	3 (20.0)	0 (0.0)	

^a^ = Fisher exact test; *p* < 0.05 was considered significant.

## Data Availability

The original contributions presented in the study are included in the article and Supplementary Material; further inquiries can be directed to the corresponding author.

## Supplementary Materials

The following supporting information can be downloaded at: https://www.mdpi.com/article/10.3390/ijerph21081063/s1, Table S1: Cases (25/75) of patients with lymphadenopathies on the CT-scan.
