# Supplementary material for: Role of COVID-19 Vaccine in the Management of Gynecologic Oncology Lymphadenopathies
Source: Int J Environ Res Public Health. 2024 Aug 14;21(8):1063. doi: 10.3390/ijerph21081063 (PMC11353838; doi:10.3390/ijerph21081063)
Supplement: Supplementary file 1 [file ijerph-21-01063-s001.zip › ijerph-3109861-supplementary.pdf]

Supplementary Materials

Table S1. Cases (25/75) of patients with lymphadenopathies on the CT-scan.

	Age	Malignancy origin	Vaccination doses and type	Vaccination-to-CT scan time (weeks)	CT-scan findings	Reason for the CT-scan	Attitude
1	54	Ovarian	2 doses Moderna	1st dose = 14<br>2nd dose = 10	High risk of malignancy lymphadenopathies	Follow-up	Additional assessment with PET-CT confirmed the presumptive diagnosis and the patient started chemotherapy treatment.
2	60	Endometrium	1 dose AstraZeneca	1st dose = 7	Low risk of malignancy lymphadenopathies	Diagnosis	Standard management, surgery was performed and anatomopathological study of the lymphadenopathies was negative for malignancy.
3	67	Endometrium	Not vaccinated before CT-scan	-	High risk of malignancy lymphadenopathies	Diagnosis	Standard management, surgery was performed and anatomopathological study of the iliac lymphadenopathies confirmed malignancy.
4	79	Endometrium	Not vaccinated before CT-scan	-	High risk of malignancy lymphadenopathies	Follow-up	Standard management, surgery was performed and anatomopathological study of the iliac lymphadenopathies confirmed malignancy.
5	81	Endometrium	2 doses Pfizer-BioNTech	1st dose = 12<br>2nd dose = 9	High risk of malignancy lymphadenopathies	Follow-up	Standard management. A CT-scan after 3 months was performed with cancer progression. Chemotherapy treatment could not be continued due to hematological side effects.

Table S1. Cases (25/75) of patients with lymphadenopathies on the CT-scan.

	Age	Malignancy origin	Vaccination doses and type	Vaccination-to-CT scan time (weeks)	CT-scan findings	Reason for the CT-scan	Attitude
6	66	Ovarian	2 doses Moderna	1st dose = 4<br>2nd dose = < 1	High risk of malignancy lymphadenopathies	Follow-up	Standard management, the patient started radiotherapy in order to ease the pain.
7	80	Uterine cervix	2 doses Pfizer-BioNTech	1st dose = 7<br>2nd dose = 3	High risk of malignancy lymphadenopathies	Follow-up	Standard management, palliative treatment was started.
8	52	Endometrium	2 doses Moderna	1st dose = 5<br>2nd dose = 1	Low risk of malignancy lymphadenopathies	Follow-up	Standard management, next year a follow-up CT-scan was performed and the lymphadenopathies had disappeared.
9	82	Endometrium	Not vaccinated	-	High risk of malignancy lymphadenopathies	Follow-up	Standard management, the patient was in palliative treatment so the committee decided clinical follow-up without imaging tests.
10	82	Ovarian	2 doses Moderna	1st dose = 9<br>2nd dose = 4	Low risk of malignancy lymphadenopathies	Follow-up	Standard management, a CT-scan was performed 3 months later with disappearance of the lymphadenopathies observed in the previous exam.
11	61	Uterine cervix	1 dose Moderna	1st dose = 2	High risk of malignancy lymphadenopathies	Follow-up	Additional assessment. A biopsy of the inguinal lymphadenopathies was performed and anatomopathological study confirmed malignancy.

Table S1. Cases (25/75) of patients with lymphadenopathies on the CT-scan.

	Age	Malignancy origin	Vaccination doses and type	Vaccination-to-CT scan time (weeks)	CT-scan findings	Reason for the CT-scan	Attitude
12	84	Ovarian	1 dose Moderna	1st dose = 3	High risk of malignancy lymphadenopathies	Follow-up	Standard management, given the clinical context, it was assumed that lymphadenopathies were secondary to cancer progression and the type of chemotherapy was changed.
13	59	Endometrium	1 dose Moderna	1st dose = < 1	Low risk of malignancy lymphadenopathies	Follow-up	Standard management. A CT-scan was performed 3 months later, demonstrating stability of lymphadenopathies.
14	66	Ovarian	1 dose AstraZeneca	4	High risk of malignancy lymphadenopathies	Follow-up	Standard management. The patient continued with chemotherapy, CT-scan was performed 3 months later, demonstrating lymphadenopathies stability.
15	68	Endometrium	Not vaccinated before CT-scan	-	Low risk of malignancy lymphadenopathies	Diagnosis	Standard management, surgery was performed and anatomopathological study of the lymphadenopathies was negative for malignancy.
16	50	Ovarian	Not vaccinated before CT-scan	-	Low risk of malignancy lymphadenopathies	Diagnosis	Standard management, surgery was performed and anatomopathological study of the lymphadenopathies was negative for malignancy.
17	77	Endometrium	Not vaccinated before CT-scan	-	High risk of malignancy lymphadenopathies	Diagnosis	Standard management, surgery was performed and anatomopathological study of the lymphadenopathies confirmed malignancy.
18	80	Endometrium	2 doses Pfizer-BioNTech	1st dose = 10<br>2nd dose = 7	Low risk of malignancy lymphadenopathies	Follow-up	Standard management. A CT-scan was performed 3 months later, demonstrating stability of lymphadenopathies.
19	60	Ovarian	Not vaccinated	-	Low risk of	Follow-up	Standard management. A CT-scan was

Table S1. Cases (25/75) of patients with lymphadenopathies on the CT-scan.

	Age	Malignancy origin	Vaccination doses and type	Vaccination-to-CT scan time (weeks)	CT-scan findings	Reason for the CT-scan	Attitude
			before CT-scan		malignancy lymphadenopathies		performed 3 months later, and showed resolution of the previous lymphadenopathies.
20	56	Uterine cervix	2 doses Moderna	1st dose = 7<br>2st dose = 3	High risk of malignancy lymphadenopathies	Follow-up	Standard management. The patient was under concomitant chemotherapy and radiotherapy treatment. A MRI was performed in order to evaluate the treatment response, demonstrating cancer progression.
21	52	Uterine cervix	Not vaccinated before CT-scan	-	High risk of malignancy lymphadenopathies	Follow-up	Standard management, the patient continued with the chemotherapy treatment, and a CT-scan performed 3 months later to evaluate treatment response showed complete radiologic response.
22	44	Ovarian	Not vaccinated before CT-scan	-	High risk of malignancy lymphadenopathies	Follow-up	Standard management. An exploratory laparoscopy was performed to assess surgical rescue after retroperitoneal lymph node relapse. Peritoneal implants were observed, so the patient started chemotherapy treatment.
23	71	Endometrium	2 doses Moderna	1st dose = 15<br>2nd dose = 8	High risk of malignancy lymphadenopathies	Follow-up	Additional assessment with an advanced imaging test control that demonstrated cancer progression with pulmonary metastasis and growth of the paraaortic and pelvic lymphadenopathies.
24	59	Ovarian	Not vaccinated before CT-scan	-	High risk of malignancy lymphadenopathies	Follow-up	Standard management, the patient continued with the chemotherapy treatment, and a CT-scan performed 6 months later demonstrated

Table S1. Cases (25/75) of patients with lymphadenopathies on the CT-scan.

	**Age**	**Malignancy origin**	**Vaccination doses and type**	**Vaccination-to-CT scan time (weeks)**	**CT-scan findings**	**Reason for the CT-scan**	**Attitude**
							complete radiologic response.
25	77	Endometrium	2 doses Pfizer-BioNTech	1st dose = 4<br>2nd dose = 1	Low risk of malignancy lymphadenopathies	Diagnosis	Standard management, surgery was performed and anatomopathological study of the lymphadenopathies was negative for malignancy.
